# *Pelodera *(syn. *Rhabditis*) *strongyloides *as a cause of dermatitis – a report of 11 dogs from Finland

**DOI:** 10.1186/1751-0147-48-18

**Published:** 2006-09-05

**Authors:** Seppo AM Saari, Sven E Nikander

**Affiliations:** 1 Department of Basic Veterinary Sciences (FINPAR), Faculty of Veterinary Medicine, P.O. Box 66, FIN-00014 University of Helsinki, Finland

## Abstract

**Background:**

*Pelodera (Rhabditis) strongyloides *is a small saprophytic nematode that lives in decaying organic matter. On rare occasions, it can invade the mammalian skin, causing a pruritic, erythematous, alopecic and crusting dermatitis on skin sites that come into contact with the ground. Diagnosis of the disease is based on case history (a dog living outdoors on damp straw bedding) with characteristic skin lesions and on the demonstration of typical larvae in skin scrapings or biopsy. *Pelodera *(rhabditic) dermatitis cases have been reported mainly from Central European countries and the United States.

**Case presentation:**

During 1975–1999, we verified 11 canine cases of *Pelodera *dermatitis in Finland. The cases were confirmed by identifying *Pelodera *larvae in scrapings. Biopsies for histopathology were obtained from three cases, and typical histopathological lesions (epidermal hyperplasia, epidermal and follicular hyperkeratosis, folliculitis and furunculosis with large numbers of nematode larvae of 25–40 μm of diameter within hair follicles) were present. The *Pelodera strongyloides dermatitica *strain from the first verified case in Finland has been maintained in ordinary blood agar in our laboratory since 1975. Light microscopy (LM) and scanning electron microscopy (SEM) studies were employed to obtain detailed morphological information about the causative agent. The rhabditiform oesophagus at all developmental stages, the morphology of the anterior end of the nematode, copulatory bursa and spicules of the male and the tail of the female were the most important morphological features for identifying *P. strongyloides*.

**Conclusion:**

These cases show that *Pelodera *dermatitis occurs in Finland, and also farther north than described earlier in the literature. This condition should be considered when a dog living outdoors has typical skin lesions situated at sites in contact with the ground as the main presenting clinical feature. The fastest and easiest way to confirm the diagnosis is to demonstrate typical larvae in skin scrapings. In uncertain cases, skin biopsy and culturing of the worms are recommended as supplementary diagnostic procedures.

## Background

*Pelodera (Rhabditis) strongyloides *(Scheider, 1860) is a small free-living saprophytic nematode that normally completes its entire life cycle in organic matter and can be very abundant there [1-3]. This species includes a particular strain referred to as *P. (R.) strongyloides dermatitica *[2], the third-stage larvae of which are capable of invading the skin, although rarely, causing dermatitis in several mammalian species, including the dog and man [2-4]. As decaying organic matter is a natural habitat of *P. strongyloides*, damp straw bedding is often present in the history of dogs suffering from *Pelodera *dermatitis. For the same reason, the hallmarks of *Pelodera *dermatitis, such as erythema, alopecia, papulocrustous skin lesions and pruritus, are usually seen one skin in contact with the ground and decaying organic matter [3,5]. The case reports of canine *Pelodera *dermatitis are predominantly from Central Europe [2] or the Midwestern United States [2,5]. Thus far, *Pelodera *dermatitis has only once been reported in the Nordic countries, almost a century ago in Oslo, Norway [6]. The morphology of *P. strongyloides*, as seen under light microscope (LM), has been provided by, for instance, Reiter [1] and Sudhaus & Schulte [2]. Wagner & Seitz [7,8] have published supplementary information to morphological observations by performing scanning electron microscopy (SEM) on adult worms. Here, we describe the clinical features of *Pelodera *dermatitis in 11 dogs in Finland. A histopathological study was performed on three cases. In addition to the morphological LM studies of parasites, we employed a SEM technique to observe the surface morphology of cultured adult worms and larvae in one skin biopsy taken from a dog with confirmed *Pelodera *dermatitis.

## Case presentation

### Geographical distribution of cases

**Figure 1:**
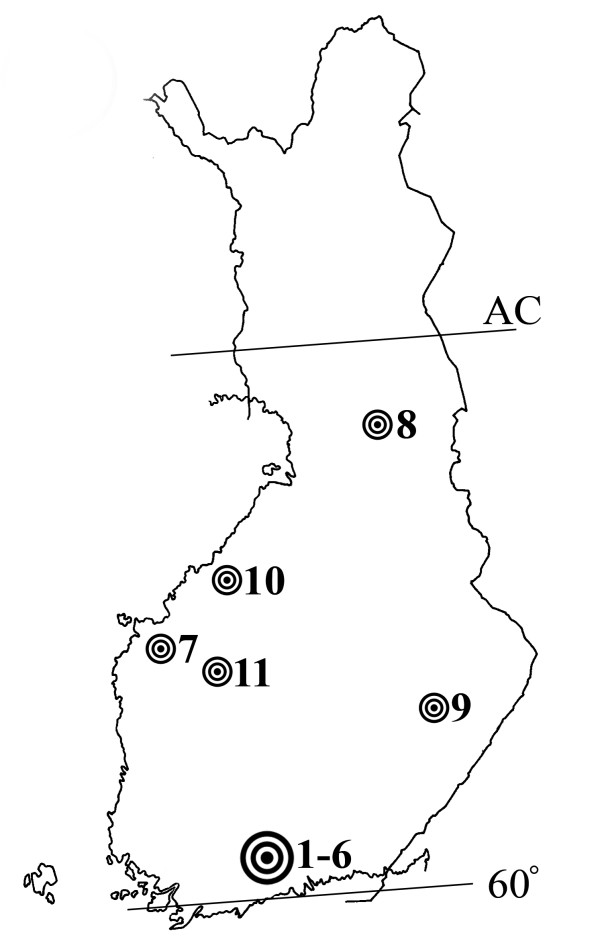
**The geographical distribution of *Pelodera *dermatitis cases verified in Finland during 1975–1999**. Note that cases 1–6 were from the same litter of German Shepherd puppies. Cases 7–11 were Finnish Hounds. All cases occurred between a latitude of 60° and the Arctic Circle (AC; 66° 33').

Before 1988, about 30 canine cases were reported in Europe and North America [2]. Since then, two additional cases have been reported, one in Germany [9] and another in Estonia [10]. During 1975–1999 we diagnosed *Pelodera *dermatitis in 11 dogs in six different locations in Finland (Figure 1). According to the maps presented by Sudhaus & Schultze [2], a majority of the European cases occurred between the latitudes of 50° and 60°, and the American cases between the latitudes of 40° and 50°. To our knowledge, the northernmost and to date the only case reported from Nordic countries is from Oslo, Norway, recorded almost a century ago by Horne [6]. He did not identify the causative agent to the species level, but the clinical course of the skin disease observed in a young Pointer, as well as the description of larvae detected in skin scraping were typical of *Pelodera *dermatitis. All of our cases occurred between the latitudes of 60° and 70°, confirming that *Pelodera *dermatitis is a skin disease that manifests farther north than described earlier.

### Clinical features

**Figure 2:**
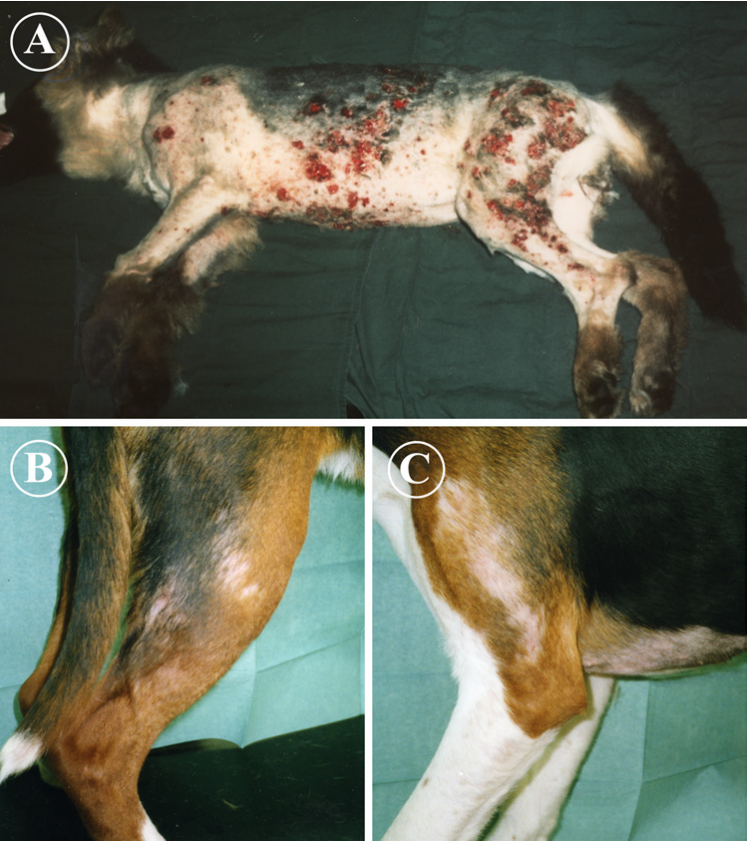
**Dogs with* Pelodera *dermatitis**. A) Severe ulcerative dermatitis and deep pyoderma are observed in a German Shepherd puppy (Case 1). Affected areas have been clipped to show the extent of the disease. B) and C) *Pelodera *dermatitis in a Finnish Hound (Case 10). Note alopecia and mild popular dermatitis with contact distribution affecting the extremities and ventral trunk.

A pruritic, alopecic, erythematous and crusting dermatitis affecting body sites in contact with the ground was a typical clinical feature in all of our cases. Six cases were seen in a litter of German Shepherd dogs (GSD)(Figure 2A), and five cases in Finnish Hounds of various ages. One of the Finnish Hounds (Case 8) had been euthanized and submitted for necropsy as a suspected case of canine scabies. The ventral abdomen, chest, perineum, distal legs, lateral shoulders and lateral thighs were most commonly affected (Figures 2A–C). Two of the GSD puppies had widespread skin lesions, sparing only the head and back. Hair clipping revealed a severe ulcerative dermatitis and deep pyoderma in these two puppies. Milder folliculitis and pyoderma were observed in the other four GSD puppies and in all Finnish Hounds.

Living outdoors and sleeping on straw bedding were common factors in all of our Finnish Hounds. This is in accordance with the findings of earlier published canine cases and with textbook data [3,5,9-12]. The GSD puppies here were reared outdoors in the breeder's fenced backyard. Although straw bedding was not used, the area contained sufficient decaying vegetation to maintain a *Pelodera *population at such a high level that six of the eight puppies of this litter got skin problems. In many textbooks, filthy conditions are cited as a requirement for the development of *Pelodera *dermatitis [3,5]. Since decaying organic matter is a typical habitat of *P. strongyloides*, removal of straw bedding from the kennel is imperative to allow successful medical treatment. Discarding moist or dirty bedding and replacing it with clean, dry bedding was the first step taken in treating our *Pelodera *dermatitis cases. All dogs were treated with ectoparasiticides. A favourable response was achieved with organophosphates (cases 1–6) and with ivermectin (cases 7, 9, 10 and 11). Oral antibiotics were used in cases with confirmed or suspected concurrent bacterial infection (cases 1–4 and case 10).

Finnish Hounds were over-represented in our cases. As an important hunting dog, it is the most popular breed in Finland (2126 registrations in 2005) [13]. Another popular breed usually kept outdoors under similar conditions is the Norwegian Elkhound (1478 registrations in 2005) [13], however, to be quite resistant to *Pelodera *infection, as it was not represented in our material at all. The skin of the Norwegian Elkhound is protected by a plush coat, whereas that of the short-coated Finnish Hound is exposed to invading *Pelodera *larvae. Our cases support earlier findings concerning the predisposing factors to *Pelodera *infection; most reported canine cases have been short-coated dogs [5].

Whether the larvae invade the hair follicles through random contact or actively seek to invade a host is unknown. The third-stage larvae of at least three other *Pelodera *species can invade the hair follicles and lachrymal glands of many rodents [2,14]. The life cycle of *Pelodera *spp. within a rodent host does not progress beyond the fourth-stage larvae. However, the parasitic phase can be advantageous to these species, especially if environmental conditions become unsuitable for completion of a free-living life cycle. During the parasitic phase, infected rodents can transfer worm populations to other rodent colonies [2,14]. A dog (as well as other domestic animals) is considered an aberrant host to *P. strongyloides*, and the inflammatory skin reaction seen during the infection is regarded as evidence of its poor adaptation as a canine parasite [2,3]. As *Pelodera *dermatitis is an apparently rare canine disease although this opportunistic parasite is very prevalent in organic matter, we have to take into consideration the possibility that, in addition to clinical cases, there are cases where the parasitic phase in a dog remains asymptomatic. Interestingly, in the GSD litter, we saw puppies with severe furunculosis and deep pyoderma and puppies with negligible or absent clinical signs. If *Pelodera *infection occurs, the immunological responses of the host play an important role in counteracting the infection and in the degree of the skin disease observed [3].

### Skin scrapings and histopathology as diagnostic tools

**Figure 4:**
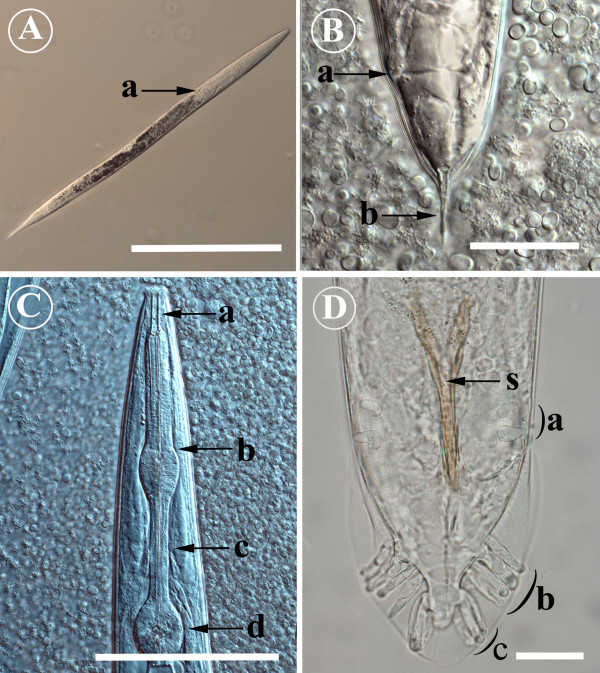
**Morphology of *Pelodera strongyloides *from light microscopy**. A) *Pelodera strongyloides *larva as seen in a skin scraping. The rhabditiform oesophagus (a, see also Fig. 4C) is the most important morphological feature to differentiate *P. strongyloides *larvae from other nematode larvae. To improve contrast in specimen, a microscope equipped with differential interference contrast (DIC) was used. Scale bar = 200 μm. B) The posterior end of female *Pelodera strongyloides*. The tail possesses a clear spine-like extension (b); a = anus. To improve contrast in specimen, a microscope equipped with differential interference contrast (DIC) was used. Scale bar = 50 μm. C) The anterior end of an adult *Pelodera strongyloides*. The light micrograph reveals a deep buccal capsule (a) and a rhabditiform oesophagus, consisting of an elongated corpus (b), followed by a distinct swelling midway through the oesophagus and the narrow isthmus (c), ending aborally with a clearly defined valvulated bulb (d). To improve contrast in specimen, a microscope equipped with differential interference contrast (DIC) was used. Scale bar = 100 μm. D) The posterior end of a male *Pelodera strongyloides*. The male has an open well-defined copulatory bursa with ten pairs of elongated papillae. Two pairs are located precloacally (a), and the remaining eight pairs posterior to the cloaca. The anterior group of postcloacal papillae consists of five papillae (b), and the posterior group (c) three papillae. Spicules (s) form Y-shaped copulatory structure. Scale bar = 20 μm.

Skin scraping is an easy, fast, inexpensive and reliable method for the diagnosis of *Pelodera *dermatitis. Diagnosis of our cases was based on clinical history and detection of typical larvae in skin scrapings. The length of larvae in skin scrapings varied from 600 to 750 μm, and the width from 30 to 40 μm (Figure 4A). The oesophagus of the larvae was of the rhabditiform type, consisting of an elongated corpus, followed by a distinct swelling midway down the oesophagus and narrow isthmus, ending aborally with a clearly defined valvulated bulb. The cuticle was distinctly transversally striated. The oral opening was surrounded by lips, but their number and arrangement could not be determined with LM.

When nematode larvae are found in skin scrapings, the differential diagnoses are few. Hookworm larvae are capable of penetrating the skin and can cause skin problems. The only hookworm species prevalent in dogs in Finland is *Uncinaria stenocephala*, but unlike the hookworm species in warmer climates, *U. stenocephala *usually does not infect dogs by the transcutaneous route. The larvae of *U. stenocephala *can be easily differentiated from *Pelodera *larvae on the basis of the rhabditiform oesophagus in the latter [2,3]. *Strongyloides stercoralis *is another nematode that can, at least theoretically, be found in skin scrapings, as dogs usually get the infection transcutaneously [3]. *S. stercoralis *is also considered a parasite of warmer climates, and, to our knowledge, it has only been diagnosed once in Finland (2006, unpublished data). As free-living nematodes are very prevalent, there is a possibility that a dog suffering from another skin disease can transitionally harbour some free-living nematodes on its skin, misleading the diagnosis when skin scrapings are taken.

**Figure 3:**
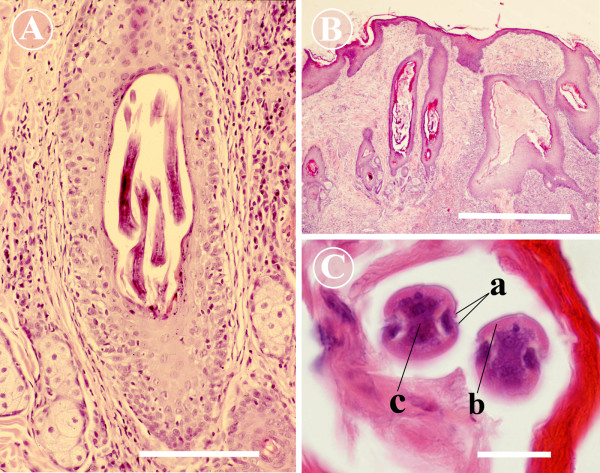
**Histopathology of *Pelodera *dermatitis**. A) A micrograph to show a superficial hair follicle distended withelongated larvae of *Pelodera strongyloides*. Lymphocytic mural folliculitis and perifolliculitis are present (Finnish Hound, Case 10). Scale bar = 200 μm. B) Histopathological findings of *Pelodera *dermatitis in German Shepherd puppy (Case 1). The epidermis is severely acanthotic. Hyperkeratosis is present in the epidermis and hair follicles. Numerous *Pelodera strongyloides *larvae can be observed within thehair follicles. Severe folliculitis, furunculosis and suppurative to pyogranulomatous cellulitis are observed. Scale bar = 1000 μm. C) Close-up of *Pelodera strongyloides *from a hair follicle of German Shepherd dog (Case 1). Cross-sections of larvae demonstrate paired lateral alae (a), platymyrian musculature (b) and hardly a discernible intestine (c). Scale bar = 20 μm.

The advantage of skin biopsy is that the pathology and the role of nematode larvae in skin disease can be reliably confirmed. Skin samples for histopathology were taken from three cases (cases 1, 8 and 10). A severely and irregularly acanthotic epidermis and epidermal and follicular hyperkeratosis were seen in all cases (Figure 3B). These changes were accompanied by folliculitis and furunculosis with discrete pyogranuloma formation. A perifollicular inflammatory reaction consisting of eosinophils, mast cells and lymphocytes was observed as well. In two cases (Finnish Hounds), lymphocytic mural folliculitis was present (Figure 3A). Large numbers of nematode larvae of 25–40 μm in diameter were seen within hair follicles. The larvae were also present in deep follicles, but were more numerous in superficial hair follicles (Figures 3A and 3B). The aforementioned histopathological changes are consistent with the descriptions in textbooks and in earlier case reports [5]. The GSD puppy of Figure 2A presented with more severe skin disease than other cases. Hair clipping revealed severe ulcerative dermatosis and lesions highly suggestive of deep pyoderma. This was confirmed in histopathology. In addition to the "classical" histopathological changes of *Pelodera *dermatitis, the puppy had extensive pyogranulomatous deep dermatitis and panniculitis.

The typical histopathological findings of *Pelodera *dermatitis are very similar to those of canine demodecosis, which is another histopathologic differential diagnosis. In histopathology, the biopsies are usually cut in 4-μm sections, and thus, only fragments of longitudinally or transversally cut larvae can be seen. These fragments of *Pelodera *larvae accompanied by typical histopathological changes can easily be misinterpreted as demodecosis. Paired lateral alae of the cuticle, the platymyarian musculature, an intestine composed of uninucleate cells and the absence of jointed appendages in *Pelodera *are features enabling differentiation even if only a few transversal sections of the parasite are observed in a biopsy sample (Figure 3C) [15].

### Culturing and morphology of *P. strongyloides*

Adult worms were not present in the skin scrapings or biopsies of any of our cases, thus, the morphological observations were based on adults obtained from the worm culture. Our *Pelodera *culture was established in 1975 by placing parasites from skin scrapings (from one GSD puppy) on ordinary blood agar in Petri dishes.

Although *Pelodera *larvae can be identified by their size and morphology, culturing of the larvae is recommended, especially in cases where identification to species level is needed and sending a sample to a parasitologist is planned. In the literature, a special medium for nematodes containing antimicrobials to prevent bacterial overgrowth has been recommended [10]. The problem with this is that these special agars are hardly ever available for the clinician at the site where the initial skin scraping is taken and *Pelodera *larvae are found. However, many small-animal practitioners do basic microbiological laboratory work in their practice, and thus, ordinary blood agar plates are usually available. Based on our experience, the ordinary blood agar plate is a suitable medium for *P. strongyloides*. The intensive bacterial growth that blooms a couple of days after the seeding of the larvae will soon fade as the new generations of *Pelodera *begin to colonize the plate. We have maintained the *Pelodera *strain originating from Case 1 for over three decades on an ordinary blood agar plate at room temperature in our laboratory. The only care that the strain has required to produce thousands of new *Pelodera *generations has been subculturing on a weekly basis.

**Figure 5:**
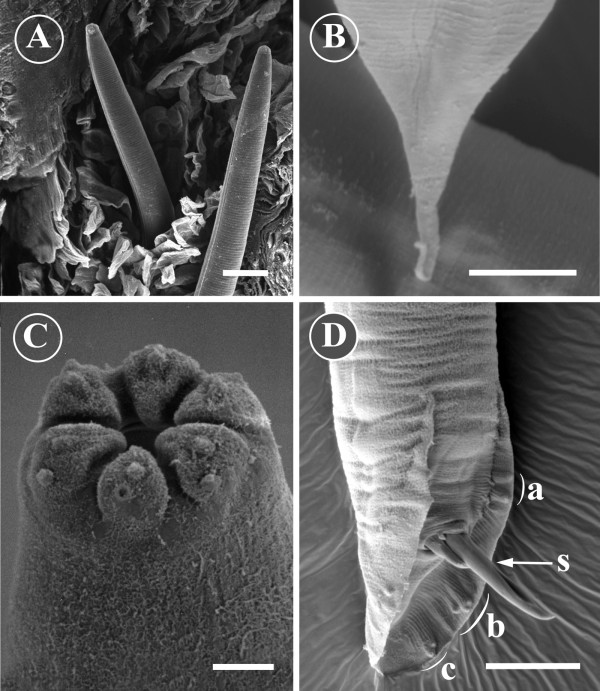
**Morphology of Pelodera strongyloides from SEM**. A) Two *Pelodera strongyloides *larvae within a hair follicle with clearly discernible lateral alae and a striated cuticle can be observed intermingling with keratin. Scale bar = 20 μm. B) The posterior end of a female *Pelodera strongyloides*. The tail possesses a clearspine-like extension. Scale bar = 10 μm. C) The anterior end of an adult *Pelodera strongyloides*. Oral opening is surrounded by six well-defined lips. Distinct papillae are present on the lips. Scale bar = 2 μm. D) The posterior end of a male *Pelodera strongyloides*. The scanning electron micrograph shows a copulatory bursa with its papillae: precloacal papillae (a) the anterior group of postcloacal papillae (b) and the posterior group (c) of three postcloacal papillae. Spicules (s) are protruding from the cloaca. Scale bar = 20 μm.

The adult worms possess many morphological features that aid identification to species level. (Figures 4B–D and 5B–D). The male bursa is typical of *Pelodera *spp. and the arrangement of bursal papillae varies from one species to another [1,2]. Similarly, the shape and dimensions of the female tail and its extension are of diagnostic interest [1-3,7,8,10]. To study these features more thoroughly, we employed a SEM technique on these worms. The worms were taken from the culture, and they and one formalin-fixed biopsy from Case 10 (1998) were routinely processed for SEM. After fixation, samples were dehydrated through a series of increasing concentrations of ethanol, followed by critical-point drying, mounting on aluminium stubs and coating with platinum. The specimens were examined under a scanning electron microscope (JEOL JMS-820) operating at 5–10 kV.

Adult worms and larvae possessed the rhabditiform oesophagus that was readily observed under LM (Figures 4A and 4C). The male had an open well-defined copulatory bursa with ten pairs of elongated papillae. Two pairs were located bilaterally precloacally, and the remaining eight posterior to the cloaca. Postcloacal papillae clearly formed two bilateral groups of papillae; the more anterior group consisted of five papillae, and the more posterior group three papillae. The spicules formed a light brown Y-shaped copulatory structure, the posterior two-thirds of which was fused (Figures 4D and 5D). The posterior end of the female was blunt, but possessed a narrow spine of 20 μm in length (Figures 4B and 5B).

In SEM, both sexes had similar anterior ends. The oral opening was surrounded by six well-defined lips with distinct papillae (Figure 5C).

In the skin biopsy processed for SEM, numerous nematode larvae were present within the hair follicles. The cuticular striation and the lateral alae were readily observed on the surface of the larvae (Figure 5A). The oral opening was surrounded by four well- defined lips, and the extensions of both lateral alae formed two additional smaller lips. Some of the larvae were surrounded by a cellular reaction consisting of red blood cells and inflammatory cells. To the best of our knowledge, this is the first time that the parasitic stages of *Pelodera *larvae have been observed *in situ *within hair follicles with the aid of SEM.

## Conclusion

These cases show that *Pelodera *dermatitis occurs in Finland. It is likely an underdiagnosed skin disease, previously having been reported only once in the Nordic countries (Norway). *Pelodera *dermatitis should be considered an important differential diagnosis when a dog kept outdoors on straw bedding has pruritic, alopecic and crusting dermatitis on skin that is in contact with the ground. The diagnosis is easy to confirm by taking a skin scraping from affected sites and recognizing typical larvae with a rhabditiform oesophagus. The worms can be easily cultured; we have maintained *P. strongyloides dermatitica *on ordinary blood agar at room temperature for over three decades in our laboratory.
